# A cross-sectional survey of the mental health needs of refugees and asylum seekers attending a refugee health clinic: a study protocol for using research to inform local service delivery

**DOI:** 10.1186/s12888-014-0356-y

**Published:** 2014-12-24

**Authors:** Frances Shawyer, Joanne C Enticott, Anne R Doherty, Andrew A Block, I-Hao Cheng, Sayed Wahidi, Graham N Meadows

**Affiliations:** Department of Psychiatry, Monash University, Clayton, VIC 3800 Australia; Southern Academic Primary Care Research Unit, School of Primary Health Care, Monash University, Clayton, VIC 3800 Australia; Mental Health Program, Monash Health, Dandenong, VIC 3075 Australia; General Medicine Program and Refugee Health, Monash Health, Dandenong, VIC 3175 Australia; Department of Medicine, Monash University, Clayton, VIC 3800 Australia; Melbourne School of Population and Global Health, University of Melbourne, Parkville, VIC 3010 Australia

**Keywords:** Refugee, Asylum seeker, Trauma, Epidemiology, Surveys, Screening, Mental disorders, Affective disorders, Anxiety disorders, Posttraumatic stress disorder

## Abstract

**Background:**

Refugees and asylum seekers have high rates of risk factors for mental disorders. In recent years, Australia has experienced a rapid increase in asylum seeker arrivals, creating new challenges for services in areas with high settlement numbers. This paper describes the design, including analytic framework, of a project set in a refugee health service in the state of Victoria, Australia, as part of their response to meeting the mental health needs of their burgeoning local population of refugees and asylum seekers. In order to assist service planning, the primary aim of this study is to determine: 1) an overall estimate of the prevalence of psychiatric disorders; 2) the specific prevalence of post-traumatic stress disorder 3) the perceived need and unmet need for mental health treatment. The secondary aim of the study is to establish matched risk ratios based on an Australian-born matched comparison group from the 2007 National Survey of Mental Health and Well-Being.

**Methods/Design:**

A cross-sectional survey is used to estimate the prevalence of psychiatric disorders in refugees and asylum seekers attending a local refugee health service. Measures include the Kessler Psychological Distress Scale-10, the Post-Traumatic Stress Disorder-8, the General-practice User’s Perceived-need Inventory together with service utilisation questions from the National Survey of Mental Health and Well-Being. Data collected from refugees and asylum seekers (*n* = 130) is matched to existing data from Australian-born residents drawn from the 2007 National Survey of Mental Health and Well-Being (*n* = 520) to produce estimates of the risk ratio.

**Discussion:**

The paper describes a prototype for what is possible within regular services seeking to plan for and deliver high quality mental health care to refugees and asylum seekers. A novel project output will be the development and dissemination of an epidemiological methodology to reliably compare mental health status in a relatively small target sample with a matched comparator group.

## Background

### Displaced persons internationally

The United Nations High Commissioner for Refugees (UNHCR) defines a refugee as a person who is outside of their country of nationality due to a well-founded fear of persecution for reasons of race, religion, nationality, particular social group membership or political opinion and is unable or unwilling to avail themselves of the protection of their country or return to it [1]. An asylum seeker is someone who is seeking protection outside their country and who may or may not be a refugee [2]. According to the UNHCR’s most recent report for 2013 [3], 51.2 million people were forcibly displaced worldwide by the end of 2013, of which 16.7 million were refugees and 1.2 million were asylum-seekers. Afghanistan was the leading country of origin of refugees (2.56 million) with the Syrian Arab Republic second (2.47 million) and Somalia third (1.12 million).

### Migrants and refugees in Australia

Australia has a very high population of immigrants, comprising 27% of the estimated resident population (ERP) (6 million) [4]. Refugees, however, comprise just 0.8% of all immigrants [5]. While refugees make for only a small proportion of the ERP, Australia has experienced a significant increase in asylum-seeker arrivals over the past few years creating new challenges in areas with high settlement numbers. Arrivals by air have been rising steadily since 2004–5, primarily the result of increased lodgments by international students seeking protection [2]; arrivals by sea have risen more dramatically.

In Australia, protection for refugees is offered through the Humanitarian program. The program includes an onshore component for people applying for protection or asylum after arrival in Australia and an offshore resettlement component for people in need of assistance overseas. In 2012–13 the number of places available was set at 20,000 (9.5% of total immigration) including 12,000 for offshore refugees and 8000 for onshore protection and the Special Humanitarian Program (SHP), this latter being for people experiencing substantial discrimination in their home country who are proposed by an immediate family member already granted protection in Australia [6]. Permanent Protection visas are for people who are already in Australia who apply for protection (or asylum) and who are found to meet Australia’s protection obligations under the Refugees Convention or the complementary protection criteria [7]. In 2012–13, 20,019 visas were granted including 60% Refugee visas, 2.5% SHP visas and 37.5% Protection and other onshore visas [6]. The top two countries of birth of recipients of visas granted offshore were Iraq (4064: 32.5%) and Afghanistan (2431: 19.4%): nearly half of visas granted were to people born in either of these two countries [6]. Onshore asylum applications have also risen substantially. In 2012–2013, 25,091 asylum seekers arrived by sea [8], an increase of over 300% on the previous year where there were 7983 boat arrivals [9]. As the demand for onshore places has increased over the past 5 years, there has been a reciprocal decrease in the number of SHP visas being granted ([6], p. 23).

This paper describes the design of a project conducted within a local Refugee Health Service in the state of Victoria as part of their response to meeting the mental health needs of their burgeoning local population of refugees and asylum seekers. This Refugee Health Service was established in 2007 and includes a hospital-based clinic and a community-based clinic. This service had an initial focus on addressing physical health needs including paediatrics, infectious diseases and complex care. However, in the context of other overstretched and inadequate local services [10], the Refugee Health Service has been expanding in order to also address the mental health needs of their clients. In order to target these finite services appropriately, it was important to ascertain the nature of their needs.

It is well understood that the majority of refugees arriving in Australia will have experienced traumatic events such as human rights abuses, persecution, violence, loss of identity and culture, and loss of family members [11]. Such experiences have a direct dose–response relationship to psychological symptoms both at individual and family levels [12]. Post migration living difficulties contribute further to mental health symptomatology [13]. Not surprisingly, the rate of long-term medical and psychological conditions is higher compared to other migrants while access to family and community support is lower [14].

From a local perspective, it seemed very evident to clinicians working in the clinics and in the community that refugees in this region often encounter serious mental health problems. However, only patchy information was available about the nature of these problems and little was known about the mental health needs of local refugees from the perspective of the refugee clients themselves [10]. In order to address these critical gaps in knowledge, an independent university-based research unit was commissioned to conduct a survey of the mental health needs of clients attending the Refugee Health Service.

### Aims of study

The primary aim of this study is to survey clients attending the community-based clinic within the Refugee Health Service in order to determine:

1. An overall estimate of the prevalence of psychiatric disorders.
2. The specific prevalence of post-traumatic stress disorder (PTSD).
3. The perceived need and unmet need for mental health treatment.

The secondary aim of the study is to compare the prevalence findings with an Australian-born matched comparison group from the 2007 National Survey of Mental Health and Well-Being (NSMHWB) in order to establish matched risk ratios. The NSMHWB, funded by the Australian Federal Government, provides information on the prevalence of selected lifetime and 12-month mental disorders based on a sample of around 8,800 Australians aged between 16 and 85 years. We hypothesised that refugees and asylum-seekers attending the clinic would show evidence of greater psychiatric morbidity relative to Australian-born residents. From a translational research perspective an aim of the study also is to pilot a suite of measures for screening use in this service and in the future, elsewhere. Hence the description of the study methods both describes the study as implemented in this setting and provides a practical description of how this set of methods could be replicated in another study and/or introduced into routine practice.

## Methods/Design

### Research design

There are two main components to the study design. Firstly, a cross-sectional survey is used to estimate the prevalence of psychiatric disorders in the Refugee Health Service (community health site). Secondly, the survey data collected from refugees is matched to existing data drawn from the 2007 NSMHWB. This matched comparison enables the prevalence of psychiatric disorders to be compared between refugee and Australian-born residents by producing estimates of the risk ratio [15].

The design for this study arose following extensive consultation with stakeholders and experts in the field and with ongoing dialogue with a steering committee set up to provide oversight to the project. The project was funded from within the budget of local services and as such the design was constrained by a clear funding limit. This necessitated efficient collection of information. While there was early consideration of using full diagnostic interviews this was rejected on basis of the complexity and resource demands of the translation task, concern that the associated burden on participants could compromise response rates with associated likelihood of substantial sampling bias, and on grounds of funding constraints. Thus, the approach taken was rather one of using chosen screening instruments or other short form instrumentation and only those considered essential in relation to the research questions. Hence, we included four brief instruments plus demographics. Our free access to the NSMHWB survey data for general research purposes through the Australian Bureau of Statistics enables us to extend the data further at no extra cost.

### Setting

Monash Health, where the project is based, is the largest public health care provider in Victoria, providing services to the South-Eastern suburbs of Melbourne and covering a population of over 750,000. The region includes the most culturally diverse municipality in Victoria and it contains disproportionally high numbers of refugees and asylum-seekers. The area receives the largest percentage of newly arrived refugees in Victoria – nearly a quarter of all arrivals to metropolitan areas - and around 8% of the nation’s refugees each year [16]. Unemployment is notably higher than average in the refugee population and median income lower [17]. A report examining the primary healthcare needs of refugees in this region [10] found that as of the 1 July 2010, there were around 19149 refugees in the region, representing around 5% (1:20) of the total population. The age of arrival showed a trend toward younger age groups with 93% under the age of 45 years and 44% under the age of 18. During 2012, approximately 50 asylum seekers were settling into the region each week [18]. The Afghani population was the largest and fastest growing group in the region making up 43% of asylum seekers for the period September-December 2012 [18].

The sole site for recruitment is the Refugee Health Service. The Refugee Health Service comprises two sites: a weekly hospital-based outpatient’s clinic and a clinic based in a community health centre. The latter site was added in 2011 as demand for services grew. Recruitment takes place in the community health site.

### Participants

Participants in the project are refugees or asylum seekers, aged between 18–85 years, and attending the Refugee Health Service (community health site) within Monash Health. Based on clinic attendance rates, it was expected that participants would be primarily from Afghanistan and Sri Lanka with a small number from Iran. Because measures are to be translated in advance, participants are required to be fluent in at least one of the major languages from these regions, including Dari/Dari-Hazaragi, Pashto, Persian/Farsi and Tamil, or English. To maximise comparability with patients at the refugee health clinic, the Australian-born sample extracted from the NSMHWB will be selected on the basis of demographics including age, gender and health service utilisation. The NSMHWB dataset is confidentialised to ensure anonymity.

### Measures

#### Demographic data

Demographic questions are important for understanding both the experience of mental health problems and need for care in the population surveyed. For example, time spent in refugee camps/detention centres [19] and separation from key family members [20] are both factors that may contribute to the development of mental health problems. Understanding the education, literacy, family structure, entitlements, occupation and visa category of the population surveyed may be important for considering how services are best delivered. Religious affiliation may also be an important consideration for service delivery, for example services seen as incompatible with religious culture may be a barrier to accessing them [21,22].

The demographic data collected include: age, gender, country of birth, ethic group, religious affiliation, month/year of arrival in Australia, time spent in refugee camps overseas and detention centres in Australia, visa category, marital status, number of children, number of children at home and their ages, family separation, languages spoken including first language, literacy, education, occupation, eligibility for a healthcare card and access to Medicare (Australia’s publically funded universal healthcare system).

#### Service utilisation

##### General health services

General health service utilisation is recorded by refugee participant answers (Yes/No) to three questions from the NSMHWB assessment:

1. *In the****past 12 months****, have you seen a general practitioner for your own physical or mental health?*
2. *In the****past 12 months****, have you been admitted overnight or longer in any hospital for a physical health problem?*
3. *In the****past 12 months****, have you seen any kind of specialist health care provider such as a specialist doctor, psychiatrist, psychologist, social worker or anyone else?*

#### Mental health services

Service utilisation with regard to mental health care will also be captured using questions from the NSMHWB assessment, for example:

*In the****past 12 months****, have you been admitted overnight or longer in any hospital for problems with your****mental health****? (that is, for things like stress, anxiety, depression or dependence on alcohol or drugs).*

Additional questions assess what kinds of mental health care participants think might be of benefit to them in the future or have/have not been of benefit to them in the past 12 months. For example:

*In the****past 12 months****, are there any kinds of help for a mental health problem that you think would have benefitted you but that you didn’t receive?* If yes*, What sort of help would have benefitted you? Who would be involved in that?*

Patterns of service use in the previous twelve months for mental health problems can be categorised into three groups: 1) requiring hospitalisation; 2) consulting a specialist health care provider such as a specialist doctor, psychiatrist, psychologist, social worker; and 3) consulting a general practitioner only.

#### Kessler-10

The Kessler Psychological Distress Scale (K10) [23] is a simple 10-item measure of psychological distress (particularly symptoms of anxiety and depression) based on a person’s emotional state during the 30 days prior to the survey interview. It is a widely used screening instrument in Australia, having been included in several state-based health surveys along with the 1997 and 2007 NSMHWB. It is also a familiar clinical tool used by GPs and other clinicians in Australia, being one of the outcome measurement tools recommended by the Department of Health for use in relation to mental health treatment funded by Medicare.

The K10 has a five-level response scale for each item ranging from 1: “none of the time” to 5: “all of the time”. The K10 can be used to indicate level of distress or likelihood of having a mental disorder. High scores indicate high levels of psychological distress or high likelihood of having a mental disorder. Different cut-off scores have been used depending on whether it is being used in clinical settings or in population surveys. The clinical cut-off score for likelihood for having distress consistent with a anxiety or depressive disorder is ≥ 20 with the range of scores for levels of severity being mild: 20–24; moderate: 25–29; and severe: 30–50 [24]. The bands applied in the NSMHWB for likelihood of having a mental disorder included low (10–15), moderate (16–21), high (22–29) and very high (30–50). In the 2007 NSMHWB, 79.6% of those with a score in the very high range had a 12-month mental disorder (assessed using the World Health Organization Composite International Diagnostic Interview) while only 10.9% of those in the low category had a 12-month mental disorder [25].

Although the Hopkins Symptom Checklist-25 (HSCL-25) [26] has been commonly used to assess anxiety and depressive symptoms in refugee populations, the K10 was selected in preference to the HSCL-25 in order to enable comparisons with the matched Australian-born sample extracted from the 2007 NSMHWB. For the purposes of our survey, the K10 had three other advantages compared to the HSCL-25. Firstly, unlike the HSCL-25 which was developed as a clinical tool, the K10 was developed specifically as a population survey instrument and has valuable psychometric properties in this regard. Secondly, the time frame of 30 days for the K10 rather than the 1 week for the HSCL-25 is a more suitable time frame for establishing a significant mental disorder. A Major Depressive Episode for example, which is likely to be a prevalent mental disorder in this group [27], requires that symptoms be present for at least 2 weeks. Thirdly, and more pragmatically, the K10 is considerably shorter than the HSCL-25, an important consideration in the design of this study.

The K10 has been translated into many languages and is being used in a large number of World Health Organization (WHO) surveys worldwide [23]. Although its validity has not been established specifically in refugee populations, it has been validated across a number of different cultural groups [28-32]. The K10 has also been used in refugee populations in Australia [33-35] and has shown good reliability and ease of use in even pre-literate participants in a sample of Afghan refugees recruited in Australia [35].

#### Traumatic events list and Post-Traumatic Stress Disorder-8 (PTSD-8)

The traumatic events list is a combined list based on the 17 items in Part 1 of the original Harvard Trauma Questionnaire [HTQ - 26], which were derived from core war-related experiences of refugee (specifically Indochinese) populations, and the 11 items from the PTSD section of the Composite International Diagnostic Interview 2.1 [36], which were based on *Diagnostic and Statistical Manual of Mental Disorders, 4*^*th*^*Edition* (DSM-IV) and *International Statistical Classification of Diseases and Related Health Problems 10*^*th*^*Edition* (ICD-10) definitions and criteria. Because there was overlap on 6 items, the total number of items on the traumatic events list is 22. Participants either read the list or have the list read to them and are asked to answer simply yes or no as to whether they, or someone close to them such as a family member, have ever experienced or witnessed any of these events.

The PTSD-8 [37] is an 8-item screening questionnaire for post-traumatic stress disorder. It was derived from Part 4 of the HTQ [26], which is a longer measure of trauma symptoms specifically designed for use in refugee populations. The PTSD-8 has acceptable performance compared to the HTQ [37]. It covers all three symptom clusters of the DSM-IV PTSD diagnosis, including four intrusion items, two avoidance items and two hypervigilance items, but excludes the diagnostically non-specific dysphoria items (e.g., sleeping difficulty, difficulty concentrating) since these overlap with depression and other anxiety disorders. Participants are asked how much each symptom has bothered then a) since the trauma and, if yes, b) in the past month. Items are answered on a fully-anchored 4-point scale (“not at all” – 1; “a little” – 2; “quite a bit” – 3; “extremely” – 4). Screening criteria for PTSD are met if there is at least one item in each symptom cluster with a score of ≥ 3.

#### General-practice Users’ Perceived-need Inventory (GUPI)

The GUPI is a very brief one-page instrument developed to assess participants’ estimation of their needs for mental health care and the meeting of those needs. It has acceptable reliability and validity [38]. Because the GUPI was developed in the context of the Australian healthcare system, an open-ended question was added to explore alternative approaches to mental health care that may be more acceptable to people from other cultures. For example, Omeri et al. [21] noted in one Afghan refugee sample that that people of this background were more likely to emphasise the importance of spiritual and community responses to trauma while mainstream mental health services such as counselling may be viewed with distrust. The additional question is:



#### Acceptability of interview

At the end of the interview, participants are asked to rate how acceptable they found the interview on a fully-anchored 7-point scale ranging from 1 = totally unacceptable to 7 = perfectly acceptable.

#### Referral activation form

A potentially important part of the assessment of mental health need is to not only collect information about indicated and expressed need for care but to also track what participants then do about that. Following consultation with senior staff at the Refugee Health Service a form was developed to record participants’ response to feedback from the survey regarding their mental health status: in particular, whether they wished to access professional assistance. Although considered unlikely, any need for emergency care associated with the interview is also recorded on the form.

#### Case file review form

Another locally developed form was devised to collect information about mental health diagnosis and treatment of participants from their medical records. We can compare this information with the survey data in order to a) obtain possible validity data for the instruments and b) assess to what extent the mental health needs of clients are being met currently.

### Translation and field testing

Because a large number of different interpreters are expected to be utilised in the project, the measures administered to participants were professionally translated into Dari, Pashto, Farsi and Tamil to support the consistency of interpreting across participants. While comprehensive cross-cultural validation of the translated instruments was beyond the scope of the study, the measures were back-translated then reviewed and field tested by cultural advisors appointed to the project. The cultural advisors, both refugees themselves, had good knowledge of mental health terminology. They included an Afghani medical doctor who was a highly experienced researcher in mental health and fluent in all project languages except Tamil and who was our primary advisor (SW); and a Sri Lankan medical doctor fluent in Tamil.

Field testing took place over 14 weeks and involved our cultural advisors trialling the measures with three informants from each of the four non-English language groups following written informed consent. Although numbers were low, we aimed to include males and females who had a similar background to the proposed study sample and who had a range of educational/literacy backgrounds. Some basic demographic information was collected from informants including age, gender, country of birth, language, education, literacy and refugee/asylum-seeker status. Informants comprised 10 males and 2 females with a mean age of 37.8 years (SD: 8.6, range: 27–50). Half the group were born in Afghanistan (*n* = 6) with the remainder from Iran or Iraq (*n* = 3) and Sri Lanka (*n* = 3). Six informants were asylum seekers on bridging visas, 5 were refugees on permanent protection visas and 1 had missing data for visa category. The group had been in Australia for an average of 7.8 months (SD: 8.6). Most of the informants indicated that they were literate in at least their primary language (*n* = 11) however a broad range of educational levels were represented (none: *n* = 3; primary school: *n* = 2; high school: *n* = 2; trade college: *n* = 3; university: *n* = 2).

The overall purpose of the field-testing procedures with these informants was to identify any major problems with the translations and terminology. This included assessing the basic intelligibility of the questions, whether there were any obvious and common cultural expressions of disorder that should be included and other contextual clarifications. Although care was taken not to change the wording of questionnaire items, on the advice of our primary cultural advisor a series of elaborations were developed so that additional explanations could be provided for participants not understanding a particular item (more likely those with low levels of education and/or literacy) and for terms likely to be ambiguous. For example, the term “nervous” (K10: items 2–3) could be further described as, “like how you might feel if faced with an examination or test” to help distinguish it from neurological conditions and depression and, for Tamil participants, from restlessness. Elaborations added for five items of the PTSD-8 were taken from the work of Durieux-Paillard, Whitaker-Clinch, Bovier and Eytan [39] and these were found to be helpful in field testing. Our primary cultural advisor had also noted that some participants with low levels of education may not be familiar with the mental health domain generally and be inclined to confuse psychiatric terminology with neurological conditions. Participants are therefore provided with the following general orientation to the project:

*Complete health involves not only physical well-being, but also mental well-being. This project is concerned with problems in mental health or well-being. Problems in mental health in this context refer to psychological or emotional problems such as feeling depressed or anxious, rather than neurological disorders of the brain, such as a stroke or seizures.*

Field testing indicated that the questionnaire items were understood by informants with the use of elaborations as required. We were advised, however, to reverse our standard practice of starting the interview with the demographic items and instead commence with the mental health questionnaires. This was because when the demographic items were delivered first, they were experienced by some participants as somewhat interrogation-like with some items having potential to be viewed with suspicion, particularly visa category. Therefore, with the exception of items related to age (required to confirm eligibility), education and language including literacy (required to help guide the interviewer on the likely extent of elaborations needed), the interview sequence was changed so that the demographic questions were last. We also included a preamble explaining the purpose of the demographic questions:

*The reason for the demographic questions is to help us understand what group-based characteristics might be associated with different mental health conditions. The questions will help us to tailor services to meet the specific mental health needs of people with different backgrounds and experiences.*

The Participant Information and Consent form was also translated into the nominated languages then reviewed by the cultural advisors. The cultural advisors were asked to sign a declaration confirming that the Participant Information Sheet/Consent form had been correctly translated into the applicable language.

### Procedure

#### Fieldwork

Clients attending the Refugee Health Service (community health site) who are eligible for the study are asked by bi-cultural staff employed by the service, through a clinic-booked or telephone interpreter if required, whether they would like to speak to a researcher about taking part in the study. Bi-cultural staff are provided with the eligibility criteria for the project. An English and translated dot-point summary of key points to be addressed is also be provided to bi-cultural staff and interpreters to assist this process.

If the client agrees, the bi-cultural staff member makes an appointment for the client to meet with one of research team who will explain the project and, if the client is willing to proceed, obtain written, informed consent. Participants are advised that their decision whether or not to take part in the survey will have no impact on their visa status or their family’s visa status. This is to help ensure participation is completely voluntary and to prevent biased responding – either exaggerating or minimising problems in the belief that this may help them obtain a permanent visa [40]. If utilized, the interpreter is asked to sign the consent form to attest to the following statement: “I have truthfully and faithfully translated the explanation of the research project in the participant’s language to the best of my skill and ability”.

Assessment interviews are arranged to best suit the participant – either immediately, at their next scheduled face-to-face appointment, or at a separate face-to-face appointment. If needed, a taxi voucher is provided for separate face-to-face appointments.

Where required, interpreters are organised by the researchers for the consent process and interview. We expect this to be required in most cases. As per local guidelines available for working with interpreters [41], an interpreter are called when:

- the participant requests one
- the interviewer can’t understand the information being conveyed by the participant
- the participant is assessed as needing an interpreter by the interviewer because of difficulty communicating in English or
- the participant prefers to speak and is more fluent in a language other than English.

While the use of translated measures and interpreters represents a compromise in terms of demonstrated reliability and validity of some of the measures used in this study, it will mean that most refugee clinic attendees will be in a position to take part in the study, substantially increasing the representativeness of the sample.

To keep costs contained, in this context interviews are conducted by Monash Health mental health staff as contribution in kind and an honours student as part of course requirements. As well, a qualified health professional is employed to conduct interviews on a casual basis as required. Training in clinical research interviewing, cultural responsiveness, working with interpreters, ethical conduct in research, the specific instruments used in the project and risk assessment and risk management is provided. Although interviewer-led scales introduces the potential for issues of shame and social status to bias results, this training is designed to foster a researcher-participant relationship that supports open responding to questions. For example, a warm, friendly professional manner is encouraged and interviewers are taught how to communicate respect and consideration. A strict informed consent process is followed with regular reminders during the interview regarding confidentiality and the participant’s right not to answer questions if they wish. Where appropriate, statements acknowledging the sensitive nature of some questions are provided. Administration including consent is estimated to take on average 45 minutes. If an interpreter is required, this time is expected to double to 90 mins [41].

#### Ethical approvals and considerations

The study is conducted in compliance with the Helsinki Declaration and has been approved by the following governing ethics committees: Monash Health Human Research Committee B (12190B) and Monash University Human Research Ethics Committee (CF13/129 – 2013000035). Written informed consent is required from all participants.

We have included a screen for post-traumatic stress disorder as this is likely to be of relatively high prevalence. However, to minimise the likelihood of distress, we have structured the interview to ensure that participants are not asked to disclose any details of any past traumas experienced including what they were. To avoid anticipatory anxiety, this will also be explained in the participant information and consent form.

In the consent form, participants are given the option to be informed if their scores indicate a likelihood of having a mental health problem based on recommended clinical cut-off scores for the K10: score of ≥ 20 [24] or the PTSD-8: at least one item in each symptom cluster with a score of ≥ 3 [37]. Participants who screen positive are encouraged to see a mental health professional for a complete assessment and, if necessary, treatment and will be provided with a copy of the applicable results to take with them. If the participant provides written consent, the interviewer can arrange a referral on their behalf such as to psychiatric or counselling services available at the Community Health service or being linked in or back to a general practitioner. As noted above, as part of the assessment of mental health need we will track both the expressed need for professional help and referrals made.

Participants, including field test informants, are given a $25.00 gift card to acknowledge the time and effort involved in participating in the survey and to offset any travel costs.

#### Matching strategy - NSMHWB Australian-born comparator group

In this study the K10 survey data collected from each refugee (*n*1 = 130) are matched with data drawn from the existing 2007 NSMHWB data set, with intent to assign where possible four Australian-born residents (*n*2 = 520) to each refugee subject. Matched sets having multiple comparators for each refugee is beneficial as it increases the study precision, yet, noted is that added precision is limited for sets having more than four comparators [42,43]. The matching strategy will involve randomly selecting NSMHWB subjects, filtered to those reporting a country-of-birth as Australia, and matched with these three factors reported by each refugee including age, gender and health service utilisation (including general, specialist and inpatient utilisation) in the previous twelve months. We will conduct an initial exploration of the NSMHWB data in order to ascertain the degree of precision possible in the matching strategy, for example delineating suitable bands for age. Matches for each refugee will be drawn from the entire Australian-born NSMHWB data set and random selection without replacement will identify 4 Australian-born residents per refugee. If it becomes apparent that reasonable matching criteria cannot find Australian-born residents for all refugee participants, then it may become necessary to exclude data from a refugee participant from the comparative NSMHWB analysis (and clearly reported as such). The K10 comparison is of primary interest here, given that the same measure was used in both surveys. Although the 2007 NSMHWB used the World Mental Health Survey Initiative version of the Composite International Diagnostic Interview (ICD-10 criteria) [44] to identify PTSD, we will also tentatively compare our findings from the PTSD-8 with Australian-born residents. It should be noted that the use of different measures to assess PTSD means that only very limited conclusions can be drawn from such a comparison.

### Power analysis

We calculated the required sample size to be 130 refugee participants, which is sufficient to detect a difference in proportion affected by a mental health condition by 0.125. This assumes that the proportion affected in the Australian-born NSMHWB sample is 0.25, power of 0.8, alpha of 0.05 and four matched Australian-born residents per refugee.

### Statistical analysis

#### Prevalence of mental disorders in refugees attending the community health refugee health service

Based on the survey data collected from refugee participants the prevalence of mental disorders can be determined using the NSMHWB likelihood bands, i.e. low: 10–15; moderate: 16–21; high: 22–29; and very high: 30–50; and also the K10 clinical cut-off scores, i.e. mild: 20–24; moderate: 25–29; and severe: 30–50. The overall frequency of K10 scores at or above the clinical cut-off (≥20) is further broken down into anxiety-dominated disorder (K10-anxiety items: 2, 3, 5, 6), depression-dominated disorder (K10-depression items: 1, 4, 7, 8, 9, 10) or a mixed disorder. Anxiety-dominant is defined as K10-anxiety ≥ 8 and K10-depression < 12; depression dominant as K10-depression ≥ 12 and K10-anxiety < 8; and mix as K10-anxiety ≥ 8 and K10 depression ≥ 12. The overall frequency of PTSD-8 scores at or above the clinical cut-off indicating a likelihood of having post-traumatic stress disorder is first calculated, then broken down using the K10 sub-groups i.e. see unpopulated Table 1 below. The demographic characteristics of the sample will also be examined and compared to those of the general population within the Monash Health catchments.

**Table 1:** **Proposed table for frequency of mental disorders in the refugee clinic sample**

		**K10-anxiety**	**K10-depression**	**K10-mixed**	**K10-none**
PTSD	Yes				
	No				

#### Refugee mental disorders comparison with Australian-born matched sample

The frequency of mental disorders will be calculated using the data from the matched Australian-born sample and Table 2 populated. A matched comparative analysis enables the prevalence of psychiatric disorders to be compared with Australian-born residents by producing estimates of the risk ratio [15]. The matched risk ratio (or relative risk) of having a mental disorder within the K10 likelihood bands as a function of refugee status will be calculated by comparing the proportions of refugees with a mental disorder to the proportions in Australian-born residents from the NSMHWB. In the matched comparative analysis, unlike in case–control studies, there is no need to account for the matching in the analysis to avoid bias. However, accounting for the matching may offer better precision; therefore we will apply a matching analysis using conditional Poisson regression [15].

**Table 2:** **Proposed table for frequency of mental disorders in the refugee clinic sample and the Australian-born matched sample**

	**Depression only (%)**	**Anxiety only (%)**	**Depression and anxiety (%)**	**PTSD** ^**1**^ **(%)**	**None (%)**
Refugee clinic sample (*n* = 130)					
Australian-born matched sample (*n* = 520)					

^1^Post-traumatic stress disorder (PTSD) screened in the refugee sample using the PTSD-8 and classified in the Australian-born matched sample using ICD–10.

## Discussion

The UNHCR entitled their 2012 report “Displacement: The New 21^st^ Century Challenge” and noted in the opening paragraph that “The year 2012 was marked by refugee crises reaching levels unseen in the previous decade…An average of 3000 people per day became refugees in 2012, five times more than in 2010” ([45], p. 11). Their most recent report noted that the level of displacement in 2013 was now the highest on record [3]. While Australia is not a major refugee-hosting country [45], the impact of this ongoing crisis has nonetheless been felt strongly at the local level in some regions as services seek to respond to the needs of this group. The paper describes a prototype for what is possible within regular services seeking to plan for and deliver high quality mental health care to refugees, as this becomes a growing issue in countries around the world. The design described here, which can be readily adjusted to accommodate new waves of refugees and changes in dominant languages, enables a rapid method of collecting key information, including demographics, prevalence of mental disorders and perceived need for mental health care, using a convenience sample of refugees from a local community health service population. The information collected can then be used to support expert opinion regarding treatment and policy responses that are most likely to be effective within the service concerned. The methodology highlights also the importance of field testing even where there are significant budgetary restraints and to be considering the order of measures as well as the item content. In research, demographic questions are very often asked first as they are typically seen as less sensitive and a useful warm-up to what are often more challenging questions. Here, however, the reverse was the case.

A novel project output will be the development and dissemination of an epidemiological methodology to reliably compare mental health status in a relatively small target sample (refugees and asylum seekers in our case) with multiple matched participants from the NSMHWB. Large national data sets, such as the NSMHWB, are potential rich sources to draw matched subject data for use in observational studies. It might be noted, for example, that the K10 has not only been used in the Australian NSMHWB, it has also being used in multiple WHO (World Health Organisation) World Mental Health Surveys across 28 countries [46]. Countries that form part of this initiative would have similar access to K10 data for comparison purposes. Sourcing the existing NSMHWB data set for ‘comparator’ subjects and then using our method herein will produce reliable risk ratio estimates for mental disorders within a study population of interest. This study will demonstrate that using existing data can be an alternative to seeking new data from a comparison group. The methodology is anticipated to be used by future researchers and health services to reduce costs and when large target samples are not viable such as existing small numbers within the community. The planned dissemination strategy includes publication of a paper specifically on the method, as well as a freely available ‘copyleft’ licence approach for key source code (e.g. STATA and SAS statistical software). Several limitations to this method should be noted. These include the use of non-contemporaneous data, potential limitations in matching criteria, and the use of different instruments to measure PTSD to calculate relative risk. As well, it remains possible that some participants’ understanding of depression and anxiety may differ from the western cultural understanding of such terms. If such terms are not fully understood, this may affect the validity of the findings. However, to limit this type of problem, the field testing directly aimed to address this issue. Feedback from our cultural advisors who conducted the field testing indicated that, with the use of elaborations for potentially ambiguous terms, questionnaire items were understood by informants. Despite these limitations, this design offers a very practical and inexpensive method for providing a meaningful estimate of relative risk.

## Abbreviations

DSM-IV: Diagnostic and statistical manual of mental disorders, 4^th^ edition
ERP: Estimated resident population
GUPI: General-practice users’ perceived-need inventory
HSCL25: Hopkins symptom checklist-25
HTQ: Harvard trauma questionnaire
ICD-10: International statistical classification of diseases and related health problems 10^th^ revision
K10: Kessler psychological distress scale
NSMHWB: National Survey of Mental Health and Well-Being
PTSD: Post-traumatic stress disorder
SHP: Special humanitarian program
UNHCR: United Nations High Commissioner for Refugees
WHO: World Health Organization
